# Early increase in serum-COMP is associated with joint damage progression over the first five years in patients with rheumatoid arthritis

**DOI:** 10.1186/1471-2474-14-229

**Published:** 2013-08-02

**Authors:** Maria L E Andersson, Björn Svensson, Ingemar F Petersson, Ingiäld Hafström, Kristina Albertsson, Kristina Forslind, Dick Heinegård, Tore Saxne

**Affiliations:** 1 R and D center, Spenshult Hospital, Oskarström, Sweden; 2 Department of Clinical Sciences, Lund, Section of Rheumatology, Lund University, Lund, Sweden; 3 Department of Rheumatology, Karolinska Institutet, Karolinska University Hospital, Huddinge, Sweden; 4 Section of Rheumatology, Department of Internal medicine Helsingborgs Lasarett, Helsingborgs Lasarett, Helsingborg, Sweden

**Keywords:** Cartilage oligomeric matrix protein, COMP, Rheumatoid arthritis, Biomarkers, Radiographic joint damage progression

## Abstract

**Background:**

Currently available biomarkers for the early tissue process leading to joint damage in rheumatoid arthritis are insufficient and lack prognostic accuracy, possibly a result of variable activity of the disease over time. This study represents a novel approach to detect an altered activity of the disease process detected as increasing serum-COMP levels over a short time and whether this would correlate with joint damage progression over the first 5 years of disease.

**Methods:**

In all, 349 patients from the Swedish BARFOT early RA study were examined. Serum-COMP was analysed by ELISA at diagnosis and after 3 months. Based on changes in serum-COMP levels, three subgroups of patients were defined: those with unchanged levels (change ≤ 20%) (N=142), decreasing levels (> 20%) (N=173) and increasing levels (> 20%) (N=34). Radiographs of hands and feet were obtained at inclusion, after 1, 2 and 5 years and scored according to Sharp van der Heijde (SHS). Radiographic progression was defined as increase in SHS by ≥5.8.

**Results:**

The group of patients with increasing COMP levels showed higher median change in total SHS and erosion scores at 1, 2 and 5 year follow-up compared with the groups with stable or decreasing COMP levels. Furthermore, the odds ratio of radiographic progression was 2.8 (95% CI 1.26-6.38) for patients with increasing COMP levels vs. patients with unchanged levels.

The group of patients with increasing COMP levels had higher ESR at inclusion but there were no baseline differences between the groups for age, gender, disease duration, disease activity (DAS28), function (HAQ), CRP, nor presence of rheumatoid factor or anti-CCP. Importantly, neither did changes over the 3-month period in DAS28, HAQ, ESR nor CRP differ between the groups and these variables did not correlate to joint damage progression.

**Conclusion:**

Increasing serum-COMP levels between diagnosis and the subsequent 3 months in patients with early RA represents a novel indicator of an activated destructive process in the joint and is a promising tool to identify patients with significant joint damage progression during a 5-year period.

## Background

Rheumatoid arthritis (RA) is a heterogeneous disease with a markedly variable course in different patients. A major challenge for the treating physician is to early upon diagnosis identify patients prone to different outcomes. In view of new developments in pharmacotherapy, it would be optimal to early identify those patients who are likely to develop a severe disease and enroll them for early aggressive treatment. On the other hand, with reliable prognostic indicators patients who have a potentially mild disease could be spared therapy associated with risks of side effects.

Disease outcome can be defined in different ways, i.e. radiographic joint damage, functional disability or mortality. Obviously these variables are interlinked, but predictors are not entirely overlapping [1]. However, radiographic outcome may be considered a key variable since it reflects a cumulative effect of inflammation, the tissue destroying process in bone and cartilage and accounts for a great deal of the disability in RA [2-4]. In search of prognostic makers to select patients for early intervention, there is little for the individual patient, while indicators that tell us that a cohort of patients has a higher risk, have been identified providing promise for future work. It is reasonably well established that some clinical and serological variables i.e. female sex, early joint erosions, presence of rheumatoid factor and/or anti-cyclic citrullinated peptide (Anti-CCP) and high inflammatory activity as measured by e.g. C-reactive protein (CRP) are negative prognostic signs, but the utility of these is limited when applied on an individual patient basis [5-7].

Another approach which is increasingly being explored is based on the assumption that release of molecules/fragments from joint tissue into the circulation reflects ongoing tissue turnover. Increased release should indicate accelerated turnover possibly with an emphasis on degradation and if persistent lead to permanent joint damage [8]. This forms the rationale for applying tissue markers to identify patients prone to rapid joint damage progression and to distinguish them from those having a more favourable prognosis.

One such biomarker is cartilage oligomeric matrix protein (COMP, thrombospondin 5). COMP is a 435-kDa homopentameric, extracellular protein primarily identified in cartilage [9,10]. Although COMP has also been identified in smaller amounts in normal ligament, meniscus, tendon, synovium, and osteoblasts as well as in scleroderma skin results from e.g. experimental arthritis clearly indicate that changes in serum levels of COMP in arthritis reflect processes in the cartilage [11]. COMP has been studied as a biological marker for diagnostic and prognostic applications as well as for evaluating treatment effects both in RA and osteoarthritis (OA) [12-17]. Thus, COMP serum levels were found to be higher in RA patients with rapidly progressive disease but the prognostic value of single measurements is limited [18-20]. In OA, similar observations have been made but interestingly in this condition changes in serum-COMP over a 1 or 3 year period seems to increase the prognostic utility [21-23]. Furthermore, it was recently shown that serum-COMP levels have a substantial genetically determined component, i.e. to as much as 40%. Thus, in some patients the high levels may not be pathological, which complicates interpretations [24].

Based on the observations that serum-COMP changes over time in individual patients with OA minimize the effects of the inter-individual variation we hypothesised that early changes in serum COMP during RA may relate to future joint damage. The present study examined this hypothesis in a well-defined prospective cohort of patients with recent onset RA. Alterations in levels of serum-COMP between diagnosis and 3 months were related to radiological outcome in hands and feet over the first 5 years.

## Method

### Patients

This study comprises 349 patients, who were included in the BARFOT (Better Anti-Rheumatic PharmacOTherapy) early RA study from November 1993 to June 1999. The BARFOT-study is a longitudinal observational multicenter study, where patients were consecutively included after having been diagnosed with RA according to the American College of Rheumatology 1987 criteria [25], provided that they had a disease duration not more than 1 year [26]. After baseline evaluation, regular follow-up assessments according to a defined study protocol were conducted. The present study included only patients where blood samples from inclusion and at 3 months follow up as well as radiographs from inclusion and at five year follow-up were available. Patients not included (n=124) did not differ from included patients regarding demographics, treatment, total SHS, serological or inflammatory markers at inclusion or at the 5 year follow-up in the observational study.

### Clinical disease assessments

Disease activity was measured by the composite index Disease Activity Score calculated on 28 joints (DAS28) [27]. Remission was defined either as DAS28 <2.6 [28,29] (EULAR remission) or according to the recently proposed EULAR/ACR remission criteria (scores on the tender joint count, swollen joint count, CRP (in mg/dl), and patient global assessment (0–10 scale) were all ≤1) [30]. Functional disability was assessed using the Swedish version of the Stanford Health Assessment Questionnaire (HAQ) [31].

### Biochemical assessments

At inclusion venous blood samples were taken before the patients started treatment with prednisolone and/or disease modifying anti-rheumatic drugs (DMARDs) and were analyzed by routine methods for erythrocyte sedimentation rate (ESR) and C-reactive protein (CRP). Rheumatoid factor (RF) was measured by an agglutination test where a positive titre was >1/20. Anti-CCP was analysed using the Immunoscan-RA ELISA CCP2 test (Euro-Diagnostica, Malmö, Sweden). A titre above 25 U/ml was regarded as positive. At inclusion and at follow-up blood samples were centrifuged at 2000 g and stored at −80°C until analysis.

Frozen serum samples from inclusion and from the follow-up after three months were thawed and analysed in parallel, i.e. all samples from one patient in the same plate, using a sandwich ELISA (AnaMar, Lund). COMP is stable in thawed and refrozen sera. Some of the samples were not thawed before analysis and some were thawed and refrozen. The detection limit of the assay is <0.1 U/L, and its intra-assay and inter-assay coefficient is < 5%. Samples were analysed in duplicate according to the manufacturer’s instructions. All analyses were carried out without knowledge of patient characteristics.

Based on changes in serum-COMP over the first 3 months and knowledge of the reference change value (RCV) of COMP, three groups of patients were defined: unchanged serum-COMP levels (change ≤ 20%) (group UnCh), increasing levels (increase > 20%) (group InCr) and decreasing levels (decrease > 20%) (group DeCr). Blood samples from the follow-up at 6 months, 1 and 2 years were also analyzed. The RCV value indicates the limit of the normal variation between intra-individual measurements. Variations 20% or less in serum COMP levels are regarded as a normal biological variation [32-35].

### Radiographic examinations

Plain radiographs of the hands and feet were taken at inclusion, after 1, 2 and 5 years. Two readers scored the radiographs independently in chronological order without knowledge of treatment assignment or clinical response. The inter-observer intra-class correlation coefficients between these two readers were above 0.9 for the erosion, joint space narrowing and total Sharp scores. Radiographic damage was scored according to the Sharp method as modified by van der Heijde (SHS), which includes the hands and feet and allows for separate calculations of a total score (range 0–448), an erosion score (range 0–280), and a joint space narrowing (JSN) score (range 0–168) [36]. The smallest detectable change in total SHS was 5.8. Radiographic progression was defined as a change of total SHS of 5.8 or more [37].

### Statistical methods

Statistical analyses were performed using PASW Statistics 18 software. All significance tests were two tailed and conducted at the 0.05 significance level. To test differences between groups Chi2 test was used for proportions and Kruskal-Wallis test with post hoc analysis for continuous variables. A logistic regression analysis was used to determine whether an increase of serum COMP can predict joint damage progress over the five first years with the different groups as categorical variables. A multiple logistic analysis was performed to assess if an increase in serum COMP, between inclusion and 3 months follow-up, of more than 20% (InCr) compared to stable levels of serum COMP, change of 20% or less (UnCh), was an independent factor of joint damage progression, i.e. change in SHS of more than 5.8. The multiple logistic analysis were controlled for gender, age, disease duration, anti-CCP, RF, DMARD and prednisolone treatment at inclusion.

### Ethical approval

Ethical approval was obtained from the Ethics Committee, Lund University, Gothenburg University, Linköpings University and Karolinska Institute (LU 398–01, Gbg 282–01, LI 01–263, KI 02–075). The study followed the guidelines from the Helsinki Declaration. Written consent from the participants was obtained.

## Results

### Serum COMP and baseline characteristics

At baseline, the median (range) serum-COMP level was 12.7 U/L (4.5-32.0).

**Figure 1:**
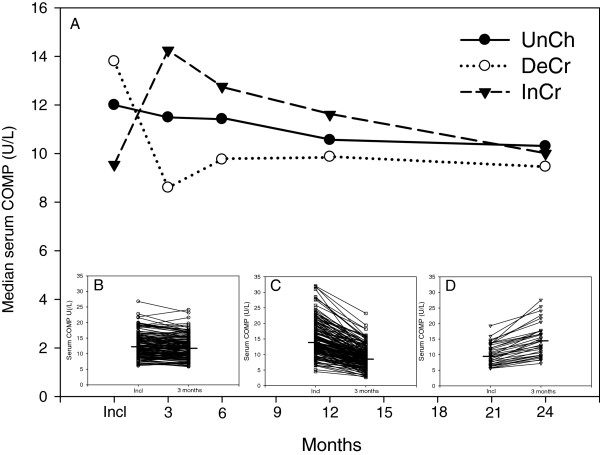
**Median value for serum-COMP over the first 2 years in the groups. (A)** shows the median value for serum-COMP over the first 2 years in the groups. The inserts show the individual changes between inclusion and the 3 month follow up in the group with unchanged serum-COMP levels (UnCh, N=142) **(B)**, the group with decreasing levels (DeCr, N=173) **(C)** and the group with increasing levels (InCr, N=34) **(D)**. The median values in the inserts are shown as a horisontal line. For further explanation, see text.

Clustering patients according to altered COMP levels over 3 months, three groups were identified: Group UnCh, of 142 (41%) patients with no change in serum-COMP, Figure 1B; group DeCr of 173 (49%) patients with decreasing serum-COMP-levels, Figure 1C; group InCr, of 34 (10%) patients with increasing serum-COMP levels Figure 1D.

Table 1 shows demographic and baseline disease variables. Except for a higher ESR in group InCr, there were no statistically significant differences between the groups. Notably, the groups did not differ regarding presence of RF or anti-CCP, two putative negative prognostic factors.

**Table 1:** Baseline characteristics at inclusion, median values (range)

	**Group UnCh (N=142)**	**Group InCr (N=34)**	**Group DeCr (N=173)**	**All (N=349)**	P-value*
Age	58 (16–83)	56 (25–83)	57 (18–84)	58 (16–84)	0.96
Gender (% women)	62	56	68	64	0.33**
Disease duration (months)	6.0 (1–13)	3.5 (1–12)	6.0 (1–21)	6,0 (1–21)	0.10
DAS28	5.4 (0.5-7.9)	5.5 (0.5-8.0)	5.1 (2.3-8.1)	5,3 (0,5-8,1)	0.06
HAQ	0.9 (0–2.6)	1.3 (0–2.3)	0.9 (0–2.5)	0,9 (0–2,6)	0.10
ESR (mm/H)	33 (2–110)	42 (2–140)	27 (2–115)	31 (2–140)	0.006
CRP (mg/L)	22.0 (4–186)	29.5 (3–159)	19.0 (4–228)	21 (3–228)	0.40
RF positive (%)	61	67	56	59	0.46**
Anti-CCP (%)	58	61	56	57	0.86**
COMP (U/L)	11.9 (6.1-26.8)	9.6 (5.6-19.3)	13.8 (4.5-32.0)	12.7 (4.5-32.0)	<0.001
Erosion score	0 (0–44)	0 (0–20)	0 (0–15)	0 (0–44)	0.68
JSN score	0 (0–40)	0 (0–15)	0 (0–33)	0 (0–40)	0.41
Total Sharp score	1 (0–84)	2 (0–34)	0.25 (0–35)	1 (0–84)	0.52

Group UnCh, patients with unchanged serum-COMP levels from inclusion to 3 months follow-up (change ≤ 20%),

Group InCr, increasing serum- COMP levels from inclusion to 3 months follow-up (increase > 20%).

Group DeCr, decreasing serum-COMP levels from inclusion to 3 months follow-up (decrease > 20%).

*Kruskal-Wallis test, ** Chi2 test.

At baseline, median serum-COMP differed between the groups being highest in group DeCr (13.8 U/L) and lowest in group InCr (9.6 U/L) and intermediate in group UnCh (11.9 U/L), overall p<0.001, Figure 1A. At three months the median serum-COMP values were lower in the DeCr group than in both the other two groups, p<0.001. At 6 months the DeCr group had still a lower median serum-COMP than both the InCr group (p<0.001) and the UnCh group (p=0.011), but there was no significant difference between the UnCh group and the InCr group. At 12 months this pattern persisted. At 24 months there were no significant differences for serum-COMP between the groups (p=0.142).

### Treatment during the 5-year period

There were no significant differences in DMARD treatment between the groups at 3 or 6 months, 1, 2 or 5 years. No patient was treated with biologics before the follow-up visit at 2 years and no difference regarding such treatment between the groups was apparent during the subsequent 3 years. Treatment with prednisolone differed between the groups. A larger number of patients in group DeCr were treated with prednisolone from inclusion to the 24 month follow-up and fewer patients in group InCr from the 3 months to the 2 year follow-up. At 5 years there were no differences in this respect between the groups.

### Radiographic outcome over 5 years

**Figure 2:**
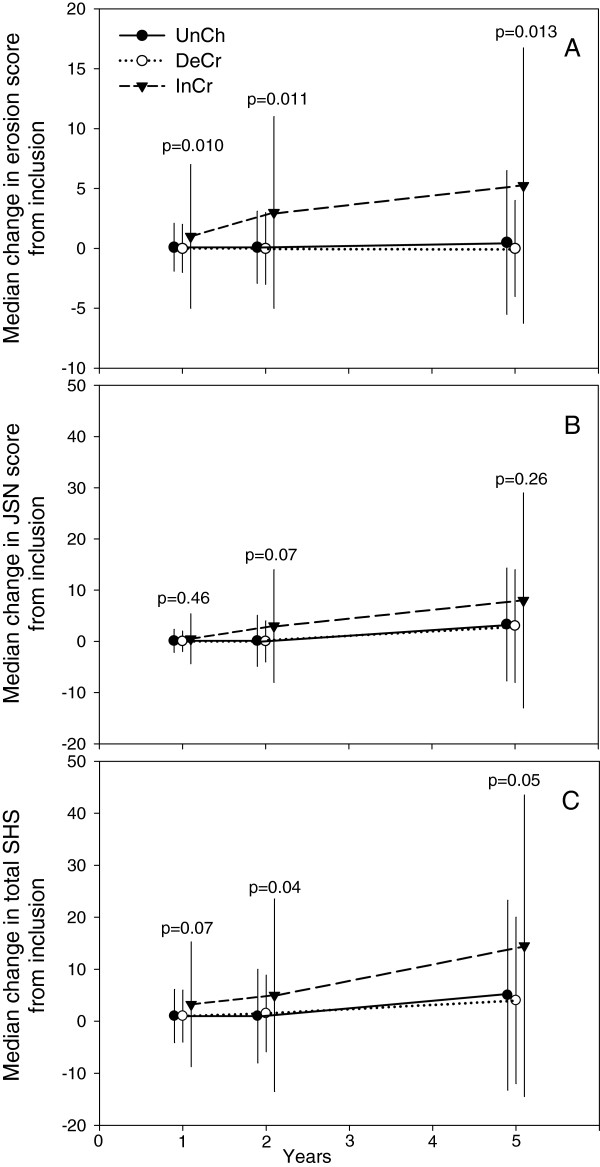
**Median change of erosion score (A), joint space narrowing (JSN) (B) and total Sharp van der Heijde score (SHS) (C) between inclusion and the 1, 2 and 5 year examinations.** Figure 2 shows median change with 75% CI (denoted by vertical lines) of erosion score **(A)**, joint space narrowing (JSN) **(B)** and total Sharp van der Heijde score (SHS) **(C)** between inclusion and the 1, 2 and 5 year examinations. Group UnCh, patients with unchanged serum-COMP levels from inclusion to 3 months follow-up (change ≤ 20%), Group DeCr, patients with decreasing serum-COMP levels from inclusion to 3 months follow-up (decrease > 20%) and Group InCr, patients with increasing serum-COMP levels from inclusion to 3 months follow-up (increase > 20%). P-values denote results of Kruskal-Wallis test between groups at 1, 2 and 5 years.

Radiographic progression after 5 years (change in total SHS score with 5.8 or more) was significantly more frequent in the InCr group, 71%, than in the other groups, 46% and 46% respectively, overall p=0.022. In the InCr group the median change in erosion score was significantly higher compared with that in the other groups at 1, 2 and 5 years, Figure 2A. Pair wise comparisons of changes in erosion score indicated significant differences between groups. Thus, group InCr showed a greater increase in erosion score than the other groups at all time points, Figure 2A. Similarly, the median change in total SHS was larger in this group but not statistically significant at all time points, Figure 2C. There were no significant differences between the groups regarding change in JSN score Figure 2B.

In a logistic regression analysis, group InCr showed an increased risk of radiographic joint damage progression at 5 years compared with group UnCh, OR 2.8 (95% CI 1.26-6.38), p=0.011. Group DeCr did not differ in this respect from group UnCh. The association remained after controlling for gender, age, disease duration, ESR, anti-CCP, RF, DMARD and prednisolone treatment at inclusion (OR 3.1 (95% CI 1.2-7.8, p=0.019). There was also an increased risk for patients positive for anti-CCP (OR 4.9 (95% CI 2.5-9.7, p<0.001).

### Clinical disease course and remission

The clinical disease activity measured by DAS28, HAQ, ESR and CRP decreased in all groups during the study period and notably there were no significant differences between the groups for changes in DAS28, HAQ, ESR or CRP between inclusion and 3 months, 1-, 2- and 5 years follow-up, respectively, data not shown. There was also no significant difference in joint count (swollen or tender) between the groups at any time point. At the five year follow-up there were no differences between the groups for these variables, except for ESR which was higher in the InCr group (p=0.02). There were no differences at five year follow-up between the groups regarding EULAR or EULAR/ACR remission. The proportion of patients in EULAR remission was 43% (UnCh group), 34% (InCr group) and 45% (DeCr group), p=0.51, respectively and the proportion in ACR/EULAR remission was 10%, 6% and 10%, respectively, p=0.81.

## Discussion

In this study we have introduced a novel approach to detect in serum samples a destructive process in one or several affected joints in patients with early RA. The background knowledge is that turnover in all the cartilages in the body is rather constant and typical for the individual, while changing when there is an activated or inhibited process in one or few cartilages. Thus, changes in serum concentrations of a molecule derived from the cartilage could be an indicator of an altered activity of the process. We have consequently assessed the relevance of altered serum concentrations of COMP, as a tool for demonstrating activity in the joint and to determine whether this could indeed be used as an indicator to signal risk for progressive joint destruction in RA. We measured the alterations in serum COMP from inclusion at early stage disease and until 3 months in a prospectively monitored early RA cohort.

By assessing this change in COMP level we could identify 3 different patient groups with unchanged, decreasing or increasing serum concentrations of COMP and compared the radiological progression of joint damage in hands and feet over a 5 year period between these 3 groups. The important main finding in support of the concept was that the small group of patients showing increased serum concentrations during the initial 3-month period, i.e. more than 20% compared to baseline, progressed significantly more than the other groups and had a more marked increase in erosion score and total SHS score at the 1, 2 and 5 year follow-up, although not significant for total SHS.

The fact that the group with increasing levels was small is not unexpected, considering that marked joint damage progression is seen only in a small proportion of patients with early RA over a 5-year period [38].

Notably, the groups did not differ regarding radiographic appearance at inclusion although the COMP levels did. Remarkably, serum COMP was lowest in the group with the largest progression but there was no association between baseline S-COMP and radiographic progression assessed according to Sharp van der Heijde at 1, 2 or 5 years (data not shown). There was also no significant associations between follow-up S-COMP at any time point and any clinical or radiographic outcome variable. These findings suggest that a single measurement does not provide a prognostic value.

Interestingly, those patients with an increasing tissue turnover activity in the early phase of the process reflected by COMP release are those who will progress the most. Other molecular indicators should be tested in a similar fashion to find out whether this is generally applicable.

It is somewhat surprising that the association between erosion score and COMP changes was more pronounced than that between COMP changes and JSN. However, the cartilage and bone processes seem closely interlinked [39]. One reason for this finding could be related to the sensitivity of the scoring system. Thus in clinical trials of potentially damage protective drugs, differences between treatment groups are often most marked for the erosion score [40].

There were essentially no differences between the groups regarding demographic characteristics and for baseline disease variables. The only difference was a somewhat higher ESR in the InCr group, which may reflect a more pronounced inflammatory process in these patients. This is not unexpected in view of the link between inflammation and joint damage progression [4,41,42]. However, there were no differences between the groups regarding changes in ESR or CRP during the study and the groups were clinically indistinguishable at five years. Furthermore, the groups did not differ regarding presence of RF or anti-CCP, which clearly suggests that the approach to evaluate prognosis regarding progression of joint damage introduced in this report adds to the armamentarium of prognostic indicators in RA.

It is important to note that the patients were treated differently with respect to glucocorticoids, i.e. more patients in the DeCr group were treated with prednisolone. Glucocorticoids are known to retard joint damage at least during the first 2 years of disease and reduce serum concentrations of COMP [43-46]. Thus the decreasing COMP levels and less progression of joint damage may be related to the prednisolone treatment in this subgroup.

A limitation to the study is that we had only access to serum samples at 0 and 3 months during the first 3 months. Analyses of samples obtained more frequently may have further strengthened the findings and importantly, would be advantageous in a clinical setting for making early treatment decisions.

An important strength of this study is the prospective setting with regular monitoring of the patients including radiographic examinations and scoring in a systematic fashion.

## Conclusion

In conclusion, we have shown that analyses of changes of serum COMP between inclusion and a 3 month follow up in patients with early RA could distinguish patients with different rates of joint damage progression over a 5 year period. Patients with increasing COMP concentrations showed the most marked progression. This approach should be further investigated, particularly by assessing the utility of examining samples obtained over a shorter time interval which potentially could improve the sensitivity and clinical feasibility.

## Competing interests

DH, TS are co-founders and minor shareholders of AnaMar, IFP is member of the board of AnaMar. The other authors declare no competing interests.

## Authors contributions

MA participated in planning the study design, gathered the data from the database, analysed the blood samples, performed the statistical analyses, and drafted the manuscript. BS participated in planning the study design, statistical analyses, data interpretation and helped draft the manuscript. IP participated in planning the study design, data interpretation and critically revised the manuscript IH participated in planning the study design, data interpretation and critically revised the manuscript. KA participated in planning the study design, read the radiographs and critically revised the manuscript. KF participated in planning the study design, read the radiographs and critically revised the manuscript. DH participated in planning the study design, data interpretation and critically revised the manuscript. TS participated in planning the study design, data interpretation and helped draft the manuscript. All authors read and approved the final manuscript.

## Pre-publication history

The pre-publication history for this paper can be accessed here:

http://www.biomedcentral.com/1471-2474/14/229/prepub
